# Construction of S-Scheme CuS/Bi_5_O_7_I Heterojunction for Boosted Photocatalytic Disinfection with Visible Light Exposure

**DOI:** 10.3390/molecules28073084

**Published:** 2023-03-30

**Authors:** Zhanqiang Ma, Wei Guo, Kaiyue Zhang, Nan Wang, Ziyue Li, Juan Li

**Affiliations:** 1 College of Agriculture, Henan University of Science and Technology, Luoyang 471000, China; 2 School of Environmental Engineering and Chemistry, Luoyang Institute of Science and Technology, Luoyang 471023, China

**Keywords:** Bi_5_O_7_I, CuS, S-scheme heterojunction, photocatalysis, inactivation of *E. coli*

## Abstract

In this paper, a novel S-scheme CuS/Bi_5_O_7_I heterojunction was successfully constructed using a two-step approach comprising the alkaline hydrothermal method and the adsorption–deposition method, and it consisted of Bi_5_O_7_I microrods with CuS particles covering the surface. The photocatalytic antibacterial effects on *Escherichia coli* (*E. coli*) were systematically examined with visible light exposure. The results suggested that the 3%-CuS/Bi_5_O_7_I composite showed the optimal antibacterial activity, completely inactivating *E. coli* (5 × 10^8^ cfu/mL) in 180 min of irradiation. Moreover, the bacterial inactivation process was scientifically described. •O_2_^−^ and h^+^ were the major active species for the inactivation of the bacteria. In the early stages, SOD and CAT initiated the protection system to avoid the oxidative destruction of the active species. Unfortunately, the antioxidant protection system was overwhelmed thereafter, which led to the destruction of the cell membrane, as evidenced by the microstructure changes in *E. coli* cells. Subsequently, the leakage of intracellular components including K^+^, proteins, and DNA resulted in the unavoidable death of *E. coli.* Due to the construction of the S-scheme heterojunction, the CuS/Bi_5_O_7_I composite displayed the boosted visible light harvesting, the high-efficiency separation of photogenerated electrons and holes, and a great redox capacity, contributing to an outstanding photocatalytic disinfection performance. This work offers a new opportunity for S-scheme Bi_5_O_7_I-based heterojunctions with potential application in water disinfection.

## 1. Introduction

Drinking-water safety is a continuing global concern. Human health can be affected by pathogenic microorganisms in drinking water. Millions of people die every year from waterborne infectious diseases. Currently, chemical oxidation technology is used for drinking-water treatment to inactivate microorganisms. Chemical oxidation disinfectants are mainly chlorine-containing preparations, such as bleaching powder, liquid chlorine, chloramine, and chlorine dioxide. Most chlorine-containing disinfectants can produce carcinogenic and mutagenic by-products during application. Ozone can also be applied to inactivate the microorganisms in drinking water, but it requires on-site preparation of expensive equipment. In 1985, Matsunaga et al. discovered that TiO_2_ loaded with Pt could inactivate *Escherichia coli* (*E. coli*) with light exposure, which introduced photocatalytic technology to the antibacterial field [1]. In the last decade, photocatalytic disinfection has attracted great attention from researchers with regard to water treatment [2,3]. Visible light-responsive materials are hot spots in the photocatalytic field for utilizing solar energy efficiently.

Bi-based photocatalysts, such as Bi_2_WO_6_, BiVO_4_, Bi_2_MoO_6_, BiOBr, and BiOI, have been extensively reported with visible light-driven photocatalytic performance. In addition, Bi_5_O_7_I with a layered crystal structure similar to that of BiOI from the [Bi_2_O_2_]^2+^ and double I layers has been discovered with a band gap of about 2.6 eV as a type of bismuth-rich BiOI. The bismuth-rich strategy has been applied to adjust the energy band structure of BiOX (X = Cl, Br, I) in order to boost the photocatalytic performance [4,5,6]. Owing to the suitable potentials of the conduction band (CB) and valence band (VB), Bi_5_O_7_I exhibits improved activities for pollutant degradation [7,8], nitrogen fixation [9,10], and CO_2_ reduction [11] compared with BiOI. However, the application of Bi_5_O_7_I is restricted by its low photoinduced carrier separation efficiency. Several modification strategies have been exploited to boost the photocatalytic performance of Bi_5_O_7_I, including vacancy engineering [12], morphology control [13], facet exposure [9], and heterojunction construction with other appropriate semiconductors [14,15,16]. Among these strategies, the construction of heterojunctions is considered to be promising for fabricating novel photocatalysts with an outstanding performance [17]. Huang et al. prepared a BiOCl/Bi_5_O_7_I 2D/3D heterostructure with a strong light capture ability and the high-efficiency separation of photoinduced carriers, contributing to the photocatalytic activity of rhodamine B (RhB) degradation being more excellent than that of than pure Bi_5_O_7_I [15]. Zhu et al. synthesized a La_2_Ti_2_O_7_/Bi_5_O_7_I heterojunction that exhibited a higher stability and better photocatalytic degradation activity of ciprofloxacin (CIP) than pure La_2_Ti_2_O_7_ and Bi_5_O_7_I [18]. It is essential to explore suitable photocatalysts coupled with Bi_5_O_7_I to promote the utilization of solar energy and to increase the quantum efficiency.

Recently, a novel step-scheme (S-scheme) heterojunction was introduced to interpret the photocatalytic mechanism [19]. An S-scheme heterojunction is achieved by using two semiconductors with staggered band structures, labeled as an oxidation photocatalyst (OP) and a reduction photocatalyst (RP) [20,21]. In consideration of the higher Fermi level (*E*_F_) of RP, the electrons (e^−^) may shift from RP to OP, and an internal electric field (IEF) is built at the interface. Due to the cooperative action of IEF, energy band bending, and Coulomb attraction, the photoinduced holes (h^+^) in the VB of RP can recombine with the photoinduced e^−^ in the CB of OP under light illumination [20,22]. As a result, the h^+^ in the OP with more positive VB potentials and the accumulated e^−^ in the RP with more negative CB potential can enhance the redox capacity of a composite. Furthermore, the separation efficiency of photoinduced carriers is also increased. CuS is a p-type sulfide-based semiconductor, and it has been explored in virtue of its broad light harvesting. However, the enhancement of photocatalytic activity is still challenging as a result of the poor quantum yield of pure CuS [23,24]. CuS-based heterojunctions, such as TiO_2_/CuS [24], Bi_2_MoO_6_/CuS [25], and Bi_2_O_2_CO_3_/CuS [26], have been constructed to promote the photocatalytic activities. Considering the interleaved energy-band structure and different *E*_F_ levels, it is possible to fabricate the S-scheme CuS/Bi_5_O_7_I heterojunctions with improved photocatalytic performance. As far as we are aware, S-scheme CuS/Bi_5_O_7_I heterojunctions have not been constructed or investigated for photocatalytic disinfection.

In this work, a CuS/Bi_5_O_7_I heterojunction was constructed using a two-step approach comprising the alkaline hydrothermal method and the adsorption–deposition method. Analyses such as XRD, SEM, XPS, UV–Vis-DRS, and PL were performed on the synthesized samples. The photocatalytic disinfection effects on *E. coli* were examined. Moreover, investigations of the *E. coli* inactivation process were carried out. The enhanced photocatalytic disinfection mechanism of the S-scheme CuS/Bi_5_O_7_I heterojunction was also proposed.

## 2. Results and Discussion

### 2.1. Characterization

**Figure 1:**
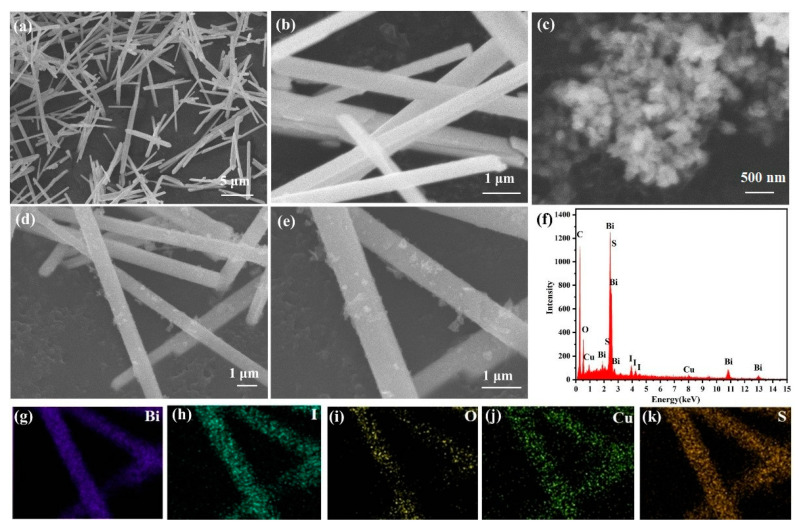
SEM images of Bi_5_O_7_I (**a**,**b**), CuS (**c**) and 3%-CuS/Bi_5_O_7_I (**d**,**e**), EDS of 3%-CuS/Bi_5_O_7_I (**f**); elemental mapping images of 3%-CuS/Bi_5_O_7_I (**g**–**k**).

SEM was used to examine the microscopic morphologies of the synthesized samples. According to Figure 1a, Bi_5_O_7_I has a the microrod structure with a diameter of ca. 100–300 nm and a length of ca. 5–10 μm. It can be also found in Figure 1b that the surface of the Bi_5_O_7_I microrods was smooth. Pure CuS was composed of approximately 100–200 nm-diameter irregular particles, which can be seen in Figure 1c. For 3%-CuS/Bi_5_O_7_I, CuS particles were deposited on the surfaces of the Bi_5_O_7_I microrods as exhibited in Figure 1d,e. Furthermore, energy disperse spectroscopy (EDS) was preformed to reveal the elemental composition and distribution of the 3%-CuS/Bi_5_O_7_I heterojunction. As shown in Figure 1f, Bi, O, I, Cu and S elements were detected in the 3%-CuS/Bi_5_O_7_I heterojunction, and the atomic ratio of Bi and I was nearly 5 (Table S1), agreeing with the molecular formula of Bi_5_O_7_I. In addition, the elemental mappings indicated that Bi, O, I, Cu, and S were evenly distributed on the surface of the 3%-CuS/Bi_5_O_7_I heterojunction (Figure 1g–k), further demonstrating that CuS particles were uniformly loaded on the surface of Bi_5_O_7_I and that the two types of materials were successfully attached.

**Figure 2:**
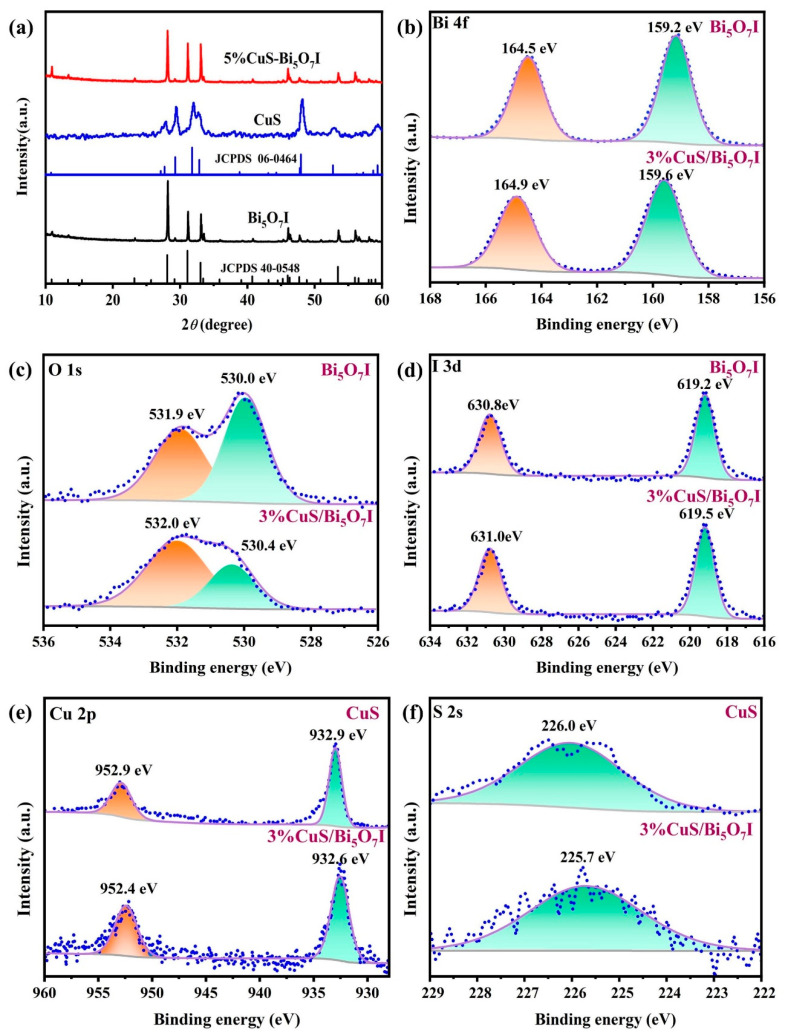
XRD patterns of synthesized samples (**a**); XPS spectra of synthesized samples: Bi 4f (**b**), O 1s (**c**), I 3d (**d**), Cu 2p (**e**), S 2s (**f**).

The crystal phases of the as-prepared Bi_5_O_7_I, CuS, and 3%-CuS/Bi_5_O_7_I composites were identified using XRD (Figure 2a). The diffraction peaks of Bi_5_O_7_I at 10.9°, 28.2°, 31.2°, 33.1°, 33.5°, 40.8°, 46.0°, 47.7°, 53.5°, 56.0°, and 58.1° can be assigned to the planes of (200), (312), (004), (204), (020), (205), (604), (224), (316), (912), and (624), respectively [27]. All the peaks are consistent with the standard card of orthorhombic Bi_5_O_7_I (JCPDS 40-0548) [28]. For pure CuS, the diffraction peaks at 27.8°, 29.3°, 31.9°, 32.7°, 48.1°, 53.0°, and 59.3° are in good accordance with the planes of (101), (102), (103), (006), (110), (108), and (116), corresponding to the hexagonal CuS (JCPDS 06–0464) [25]. After coupling Bi_5_O_7_I with CuS, the characteristic peaks of CuS cannot be identified in the 3%-CuS/Bi_5_O_7_I, as a result of the low amount of CuS.

The element composition of the 3%-CuS/Bi_5_O_7_I composite was demonstrated using XPS. In Figure S1 (see Supplementary Materials), the co-existence of Bi, O, I, Cu, and S elements can be detected in the survey spectra. In the Bi 4f high-resolution XPS spectrum in Figure 2b, two peaks at 159.6 and 164.9 eV are in line with the typical binding energies of Bi 4f_7/2_ and Bi 4f_5/2_, respectively [29]. In Figure 2c, two symmetric peaks were created by fitting the asymmetric O 1s XPS spectrum. The peak at 530.4 eV corresponds to oxygen in the Bi-O of Bi_5_O_7_I, and the peak at 532.0 eV is ascribed to the absorbed -OH group or H_2_O [15,30]. The two peaks of I 3d at 619.5 and 631.0 eV (Figure 2d) are assigned to I 3d_5/2_ and I 3d_3/2_, respectively [27]. Moreover, the corresponding peaks of Cu 2p_3/2_ and Cu 2p_1/2_ can be seen at 932.6 and 952.4 eV (Figure 2e), respectively, relative to the Cu(II) species in CuS [31]. A broad peak of S 2s at 225. 7 eV (Figure 2f) can be associated with the sulfide ions of CuS. In addition, the change in the electron density can be determined by examining the shift of the binding energy in XPS [32,33]. Compared with pure Bi_5_O_7_I, the Bi 4f, O 1s and I 3d peaks of the 3%-CuS/Bi_5_O_7_I composite shifted to a higher binding energy slightly, implying the decrease in the electron density. However, the binding energy of the S 2s and Cu 2p peaks in the 3%-CuS/Bi_5_O_7_I composite show a negative shift compared to pure CuS, demonstrating an increase in the electron density. The XPS results indicate that the electrons could migrate from Bi_5_O_7_I to CuS after the formation of the CuS/Bi_5_O_7_I heterojunction.

**Figure 3:**
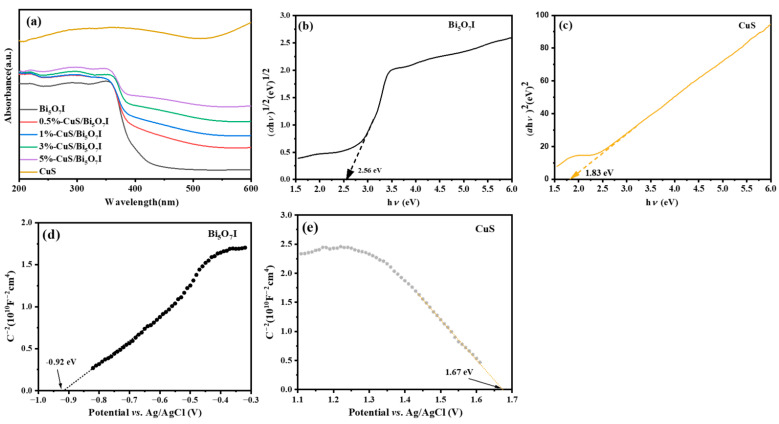
UV–Vis diffuse reflectance spectra of synthesized samples (**a**), the corresponding band gap of Bi_5_O_7_I (**b**) and CuS (**c**) from Tauc plots, M-S plots of Bi_5_O_7_I (**d**) and CuS (**e**).

As exhibited in Figure 3a, the optical characteristics of the materials were determined using a UV–Vis diffuse reflectance spectrometer. The absorption edge of Bi_5_O_7_I is at about 450 nm and the corresponding band gap energy is 2.56 eV, calculated using the Kubelka–Munk theory (Figure 3b). As for pure CuS, the outstanding visible light absorption could be identified, and the corresponding band gap energy is 1.83 eV, which is displayed in Figure 3c. The absorption intensity of the CuS/Bi_5_O_7_I composite is enhanced and unlike pure Bi_5_O_7_I, a red-shift absorption edge could be observed. The results indicated that the CuS particles deposited on the Bi_5_O_7_I microrods are in favor of the visible light harvesting.

The Mott–Schottky test was performed to study the semiconductor type, as well as the band-edge potentials, of Bi_5_O_7_I and CuS. The M-S plot of Bi_5_O_7_I with a positive slope of the linear portion can be seen in Figure 3d, indicating that Bi_5_O_7_I is an n-type semiconductor. As for CuS in Figure 3e, it is a p-type semiconductor as a result of the negative slope in the linear portion. The flat band potentials (*E*_fb_) of Bi_5_O_7_I and CuS are valued to be −0.92 and 1.67 eV (*vs*. Ag/AgCl), respectively. Based on *E*_NHE_ = *E*_Ag/AgCl_ + 0.197 [18], the *E*_fb_ values of Bi_5_O_7_I and CuS can be transformed to be −0.72 and 1.87 eV (vs. NHE), respectively. The CB potential (*E*_CB_) of the n-type semiconductor is 0.1 eV more negative than *E*_fb_ [34,35], so the *E*_CB_ value of Bi_5_O_7_I is −0.82 eV. For p-type semiconductor, the VB potential (*E*_VB_) is 0.1 eV more positive than *E*_fb_, and the *E*_VB_ value of CuS is 1.97 eV [36]. On account of *E*_CB_ = *E*_VB_ − *E*_g_, the *E*_VB_ value of Bi_5_O_7_I and the *E*_CB_ value of CuS are 1.74 and 0.14 eV, respectively.

**Figure 4:**
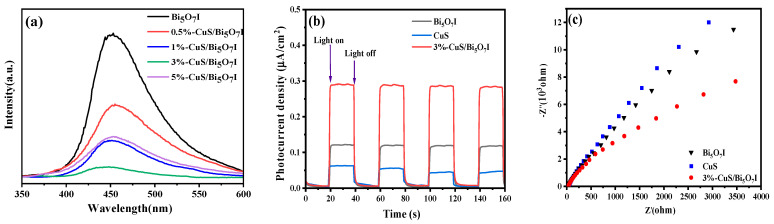
PL spectra (**a**), transient photocurrent response (**b**) and EIS (**c**) of synthesized samples.

The separation efficiency of the photoinduced carriers could be evaluated by examining the PL spectra, transient photocurrent response and EIS. Generally, lower photoinduced carrier recombination rate leads to a decreased PL emission intensity. As depicted in Figure 4a, pure Bi_5_O_7_I exhibited a remarkable fluorescence emission from 350 nm to 600 nm. For the CuS/Bi_5_O_7_I composites, the PL intensity was diminished, suggesting that the recombination of the photoinduced carriers was inhibited. Furthermore, it was noticeable that the PL intensity of 3%-CuS/Bi_5_O_7_I was the lowest. As illustrated in Figure 4b, the photocurrent intensity of 3%-CuS/Bi_5_O_7_I is about 0.28 μA/cm^2^, which is higher than that of Bi_5_O_7_I, suggesting that efficient electron–hole separation was achieved after the introduction of CuS. In addition, 3%-CuS/Bi_5_O_7_I exhibited a smaller arc radius than Bi_5_O_7_I (Figure 4c), which is helpful for transmitting and separating photogenerated carriers. In summary, the CuS/Bi_5_O_7_I composite could separate the photoinduced carriers with a high efficiency after the introduction of CuS, and this can be contributed to an improved photocatalytic disinfection performance.

### 2.2. Photocatalytic Disinfection Activity

**Figure 5:**
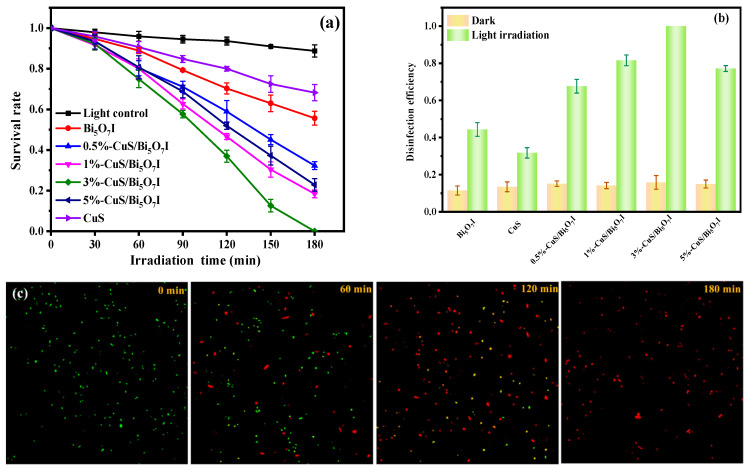
Photocatalytic disinfection activities of synthesized samples for *E. coli* (**a**), photocatalytic disinfection efficiency of synthesized samples in dark and with light illumination (**b**), the fluorescence microscope images of stained *E. coli* during the photocatalytic disinfection process of 3%-CuS/Bi_5_O_7_I (**c**).

The antibacterial performances were determined by observing the inactivation of *E. coli* with visible light exposure. As presented in Figure 5a, a light control experiment without photocatalysts was conducted to exclude the impact of light on *E. coli*. It was apparent that the survival rate of the bacteria did not noticeably reduce after the light control experiment, indicating that the effect of the visible light on the *E. coli* was insignificant. When the *E. coli* solution and photocatalysts were mixed and exposed to visible light, all the synthesized samples exhibited antibacterial performance. In particular, the antibacterial performances of the CuS/Bi_5_O_7_I composites were superior to those of CuS and Bi_5_O_7_I. Furthermore, the loading amounts of CuS had an impact on the photocatalytic antibacterial rates of the CuS/Bi_5_O_7_I composites. The photocatalytic antibacterial performance first boosted with an increase in CuS in the CuS/Bi_5_O_7_I composites until the mass ratio reached 3 wt%. However, it gradually declined with a further increase in the CuS amount, demonstrating the critical role of CuS in adjusting the antibacterial performance of CuS/Bi_5_O_7_I composites. The results of the antibacterial activities in the dark are displayed in Figure 5b. The antibacterial performances of the synthesized samples in the dark were inadequate compared to the performances of those exposed to visible light, demonstrating the production of synergistic effects with photocatalysts and visible light on the inactivation of bacteria.

As detected in Figure 5a, the 3%-CuS/Bi_5_O_7_I composite displayed the optimal performance and could inactivate all the *E. coli* within 180 min. To further evaluate the inactivation procedure of *E. coli* with the 3%-CuS/Bi_5_O_7_I composite, fluorescence microscopy was performed with stained *E. coli*. The *E. coli* was stained with PI and SYTO9 simultaneously. PI can penetrate through the broken cytomembranes and interact with DNA to label dead cells with red fluorescence. SYTO9 can be applied to label live cells with a green fluorescence because it penetrates intact cytomembranes and interacts with DNA [37]. According to Figure 5c, all the cells showed green fluorescence before the light irradiation, suggesting the presence of live bacteria with an intact cytomembranes. After prolonging the light exposure, the green fluorescence reduced progressively, while the red fluorescence increased. After 180 min of visible light exposure, only red fluorescence could be observed, implying that all the cytomembranes were damaged.

### 2.3. Inactivation Process of E. coli

#### 2.3.1. Investigation of Active Species

**Figure 6:**
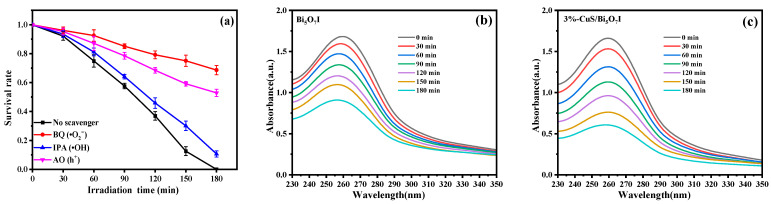
Photocatalytic disinfection efficiency of 3%-CuS/Bi_5_O_7_I with different scavengers (**a**), the absorption spectra of NBT solution with Bi_5_O_7_I (**b**) and 3%-CuS/Bi_5_O_7_I (**c**) with visible light exposure.

The experimental trapping of active species was carried out in the photocatalytic disinfection process with the 3%-CuS/Bi_5_O_7_I. Ammonium oxalate (AO), isopropanol (IPA) and p-benzoquinone (BQ) were designated as the h^+^, •OH and •O_2_^−^ scavengers, respectively. First, the toxic effects of the scavengers on the *E. coli* cells were explored. As can be seen in Figure S2, there was no discernible decline in the survival rate of *E. coli*, suggesting that the toxic effects of scavengers could be negligible. It is noticeable in Figure 6a that the photocatalytic antibacterial activities of 3%-CuS/Bi_5_O_7_I distinctly decreased after the addition AO or BQ, while the effect of IPA was tiny. Overall, the findings demonstrated that the major active species were •O_2_^−^ and h^+^ in the photocatalytic disinfection process of 3%-CuS/Bi_5_O_7_I. To further explore the production of •O_2_^−^ by Bi_5_O_7_I and 3%-CuS/Bi_5_O_7_I, nitroblue tetrazolium (NBT) was taken as an •O_2_^−^ probe agent. It is widely acknowledged that •O_2_^−^ can react with NBT to produce formazan, implying that a decline in the representative absorption peak intensity of NBT at 259 nm can be observed with the generation of •O_2_^−^ [38,39]. According to Figure 6b,c, the peak intensity of NBT for 3%-CuS/Bi_5_O_7_I decreased more notably than that for Bi_5_O_7_I, suggesting that more •O_2_^−^ could be produced by 3%-CuS/Bi_5_O_7_I.

#### 2.3.2. Activities of the Antioxidant Enzymes

**Figure 7:**
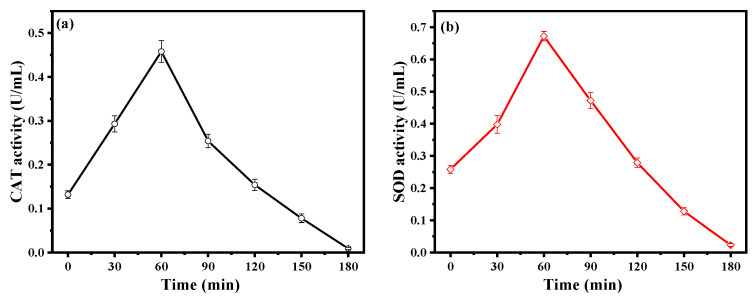
CAT (**a**) and SOD (**b**) activities of *E. coli* during the photocatalytic disinfection process of 3%-CuS/Bi_5_O_7_I.

CAT and SOD are two valued antioxidant enzymes in the *E. coli* cells, and they can protect the cell from the stress of active species by transforming them into water and oxygen [40,41]. As illustrated in Figure 7a,b, the activities of CAT and SOD with the 3%-CuS/Bi_5_O_7_I increased within the first 60 min of irradiation, indicating a defensive behavior whereby the oxidative damage of active species was resisted. However, reductions in SOD and CAT activities were detected because the overwhelmed defense capabilities of SOD and CAT were overwhelmed by the excessive attack and continuous accumulation of active groups. The collapse of the antioxidant protection system was accountable for the subsequent oxidative injury to the membrane of the *E. coli* cells.

#### 2.3.3. Microstructure Changes of *E. coli* Cell

**Figure 8:**
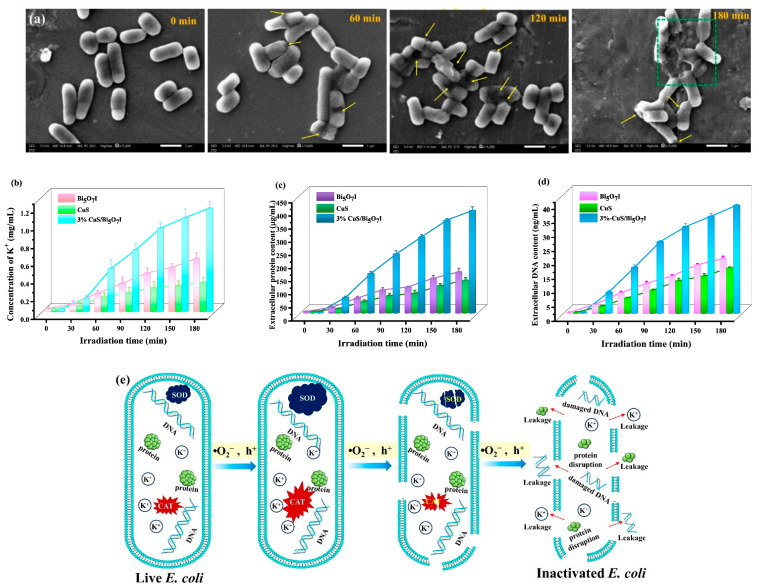
SEM images of *E. coli* cells during the photocatalytic disinfection process of 3%-CuS/Bi_5_O_7_I (**a**); concentration of leaked K^+^ (**b**), protein (**c**) and DNA (**d**) from *E. coli* during the photocatalytic disinfection process; illustration summarizing the proposed inactivation process of *E. coli* (**e**).

An evaluation of microstructure changes is helpful to understand the apoptosis process of *E. coli*. SEM was selected to reveal the microstructure changes in the *E. coli* cells treated with the 3%-CuS/Bi_5_O_7_I and visible light exposure (Figure 8a). The untreated *E. coli* cells displayed a regular short rod-like shape with blunt rounded ends. After irradiation for 60 min, some *E. coli* cells exhibited surface depressions (yellow arrows) due to the damage of their cytomembranes and the leakage of cellular components. As the irradiation time increased, more damaged *E. coli* cells could be detected and the degree of surface depressions increased. Finally, all *E. coli* cells were destroyed and the collapsed cells were liable to adhesion and aggregation (green box).

#### 2.3.4. Leakage of Intracellular Components

The determination of the cell membrane permeability can contribute to the further understanding of the inactivation process of bacteria. When the active species destroyed the cell membrane, the intracellular components could be leaked into the external environment [42,43]. K^+^, proteins, and DNA were selected as the representatives of the intracellular components in order to analyze the bacterial cell membrane permeability. It is vitally important for K^+^ to maintain cell osmotic pressure, assist with the synthesis of proteins, and balance alkalinity [41]. In Figure 8b, the leakage of K^+^ progressively increased with the increase in the irradiation time. Furthermore, the extracellular K^+^ concentration with the 3%-CuS/Bi_5_O_7_I heterojunction (1.17 mg/mL) was considerably greater than that with CuS (0.33 mg/mL) or Bi_5_O_7_I (0.60 mg/mL) after 120 min of irradiation. Similar results were obtained for the extracellular protein content (Figure 8c). Typically, proteins can recover and regrow after the injury via bacterial repair mechanisms [44]. Therefore, this is lethal to the bacteria with the injury and a loss of the nucleic acid [45]. Figure 8d proves that DNA was increasingly released during the photocatalytic disinfection process and the extracellular DNA content with the 3%-CuS/Bi_5_O_7_I heterojunction (38.5 ng/mL) was greater than that with CuS (16.2 ng/mL) or Bi_5_O_7_I (19.5 ng/mL) under illumination for 120 min. The results indicated a change in the cell membrane permeability and the leakage of intracellular components. Furthermore, the extent of the damage for cell membrane with 3%-CuS/Bi_5_O_7_I was more severe than that with CuS or Bi_5_O_7_I.

Figure 8e presents an illustration of the proposed inactivation process of *E. coli*. First, the cell was attacked by the photoinduced active species and the antioxidant enzymes, including SOD and CAT, initiated the protection system to avoid the oxidative damage. With the increasing oxidative stress, the antioxidant protection system became overwhelmed. Then, the accumulated active species destroyed the cell membrane. Eventually, apoptosis was triggered by the release of internal components through the ruptured cell membrane.

### 2.4. Mechanism of Improved Photocatalytic Activity with CuS/Bi_5_O_7_I Heterojunction

**Figure 9:**
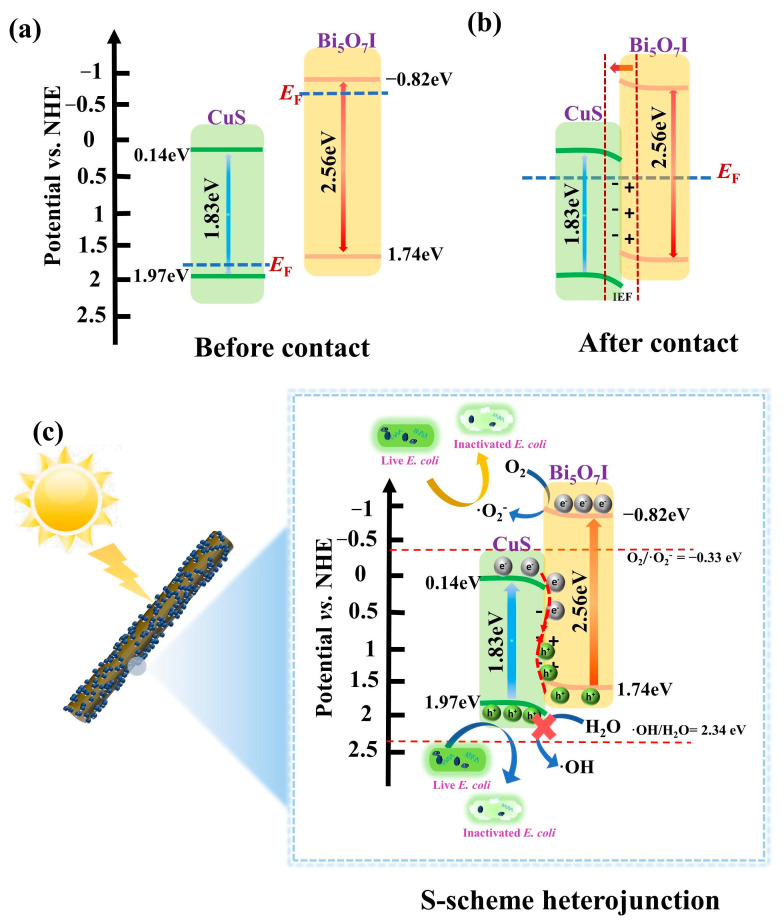
Schematic diagram of energy-band positions of CuS and Bi_5_O_7_I before contact (**a**) and after contact (**b**) and effect of S-scheme charge transfer mechanism of CuS/Bi_5_O_7_I composite on *E. coli* with visible light exposure (**c**).

The mechanism of the improved photocatalytic effects of the CuS/Bi_5_O_7_I heterojunction on *E. coli* inactivation was suggested, and it is depicted in Figure 9. Based on the previous results, CuS and Bi_5_O_7_I have CB values of 0.14 eV and −0.82 eV (vs. NHE), respectively, while their VB values are 1.97 eV and 1.74 eV. (vs. NHE), respectively. On account of the p-type nature of CuS and the n-type nature of Bi_5_O_7_I, the *E*_F_ level of CuS is close to VB, while the *E*_F_ level of Bi_5_O_7_I is near CB [36,46]. According to Figure 9a, the *E*_F_ level of Bi_5_O_7_I is higher than that of CuS. When Bi_5_O_7_I was combined with CuS, the electrons migrated from Bi_5_O_7_I with a higher *E*_F_ to CuS with a smaller *E*_F_, resulting in the formation of IEF [36,47], which can be seen in Figure 9b. The IEF can cause the accumulation or depletion of free charge carriers near the surface and result in the foundation of a well-developed heterojunction interface. The energy-band edges of Bi_5_O_7_I are bent upward toward the interface continuously, and those of CuS are bent downward towards the interface [48,49]. Eventually, the *E*_F_ levels of CuS and Bi_5_O_7_I are aligned at the same level.

With visible light exposure, Bi_5_O_7_I and CuS can be excited simultaneously. For both semiconductors, photogenerated e^−^ transferred from VB to CB, leaving h^+^ on VB. As shown in Figure S3, the CB potential of Bi_5_O_7_I is (−0.82 eV) is more negative than that of CuS (0.14 eV). As a result, e^−^ in the CB of Bi_5_O_7_I could transfer to that of CuS. Comparably, the h^+^ in the VB of CuS could shift to that of Bi_5_O_7_I. Nevertheless, it is impossible for the gathered e^−^ in the CB of CuS to react with O_2_ and generate •O_2_^−^ due to the CB potential of CuS (0.14 eV) being more positive than that of O_2_/•O_2_^−^ (−0.33 eV vs. NHE) [50]. Accordingly, the typical type II mechanism of charge transfer is obviously contrary to the results of the active species, and it is unsuitable for the CuS/Bi_5_O_7_I heterojunction. Based on the afore-mentioned analysis, an S-scheme heterojunction structure of CuS/Bi_5_O_7_I was suggested and the conceivable mechanism is depicted in Figure 9c. The downward-bending band permits e^−^ to flow out easily while inhibiting h^+^. In contrast, h^+^ can move along the upward-bending band, while e^−^ cannot [51]. Due to the cooperative action of Coulomb attraction, band bending and IEF, the e^−^ from the CB of CuS may facilely recombine with the h^+^ from the VB of Bi_5_O_7_I [22,52,53]. The CB potential of Bi_5_O_7_I (−0.82 eV) is more negative than that of O_2_/•O_2_^−^ (−0.33 eV vs. NHE), and the remaining e^−^ is competent for reducing O_2_ to •O_2_^−^. In the current study, the generated •O_2_^−^ and the h^+^ from the VB of CuS destroyed the *E. coli* cell membrane, leading to the release of cellular components. In this manner, the efficient separation of photoinduced carriers and an enhanced redox capacity can be achieved, which contribute notably improving the photocatalytic disinfection performance.

## 3. Experimental Section

### 3.1. Preparation of Photocatalysts

The details of the used chemicals and reagents can be found in the supporting information. The alkaline hydrothermal method was applied to prepare Bi_5_O_7_I. As is typical, 2 mmol Bi(NO_3_)_3_·5H_2_O and 2 mmol KI were dispersed in 30 mL distilled water by stirring, respectively. The KI solution was then added drop by drop to the Bi(NO_3_)_3_ solution. After continuous stirring for 1 h, the pH value of the above solution was adjusted to 13 by adding 3 mol L^−1^ NaOH. Next, the solution was poured into an autoclave and heated at 160 °C for 12 h. After washing with ethanol and distilled water, a white precipitate was obtained. Lastly, the product was dried and collected.

**Figure 10:**
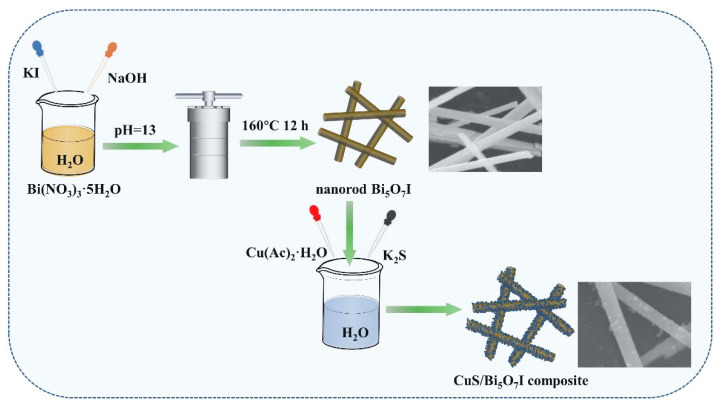
Schematic diagram of the preparation of CuS/Bi_5_O_7_I composite.

The adsorption–deposition method was adopted to synthesize CuS/Bi_5_O_7_I composites. First, a solution of 500 mg Bi_5_O_7_I was prepared in 120 mL deionized water, and the desired amounts of 0.1 mol L^−1^ Cu(CH_3_COO)_2_ solution were added dropwise. The mixture was sonicated and stirred for 1 h. Subsequently, 0.1 mol L^−1^ K_2_S solution with the same volume of Cu(CH_3_COO)_2_ solution was mixed with the above mixture under constant stirring for 2 h. Eventually, the products were washed and dried. Based on the above process, the composites were fabricated and marked as x-CuS/Bi_5_O_7_I, with x representing the mass ratio of CuS to Bi_5_O_7_I (0.5%, 1%, 3%, and 5%). Correspondingly, pure CuS was prepared correspondingly without the addition of Bi_5_O_7_I. A schematic diagram of the preparation of the CuS/Bi_5_O_7_I composites is exhibited in Figure 10.

### 3.2. Characterization

The characterization of the synthesized materials is clarified in the supporting information.

### 3.3. Photoelectrochemical Measurement

Electrochemical tests, such as transient photocurrent response, electrochemical impedance spectroscopy (EIS), and Mott–Schottky (M-S) plots, were studied using a three-electrode system on a CHI760E electrochemical workstation. The selected electrodes and measurement parameters are described in the supporting information.

### 3.4. Determination of Photocatalytic Disinfection Performance

To evaluate the photocatalytic disinfection performance of the as-prepared materials, *E. coli* (ATCC 8739) was taken as the model bacterium. All microbiological-related apparatuses and operations should be kept sterile for the bactericidal experiments. A single *E. coli* colony was taken from a flat plate and inoculated in a Luria–Bertani (LB) liquid medium to culture overnight at 37 °C. Next, 1% of the inoculation amount was transferred to a fresh LB liquid medium and cultured at 37 °C for 3 h. After centrifugation at 8000 rpm for 5 min, the bacteria were collected and dispersed in saline. Typically, the photocatalytic disinfection investigation was conducted with a 100 mL *E. coli* solution (5 × 10^8^ cfu/mL) treated by adding 20 mg photocatalyst. As a light source, a 300 W Xe lamp with a 420 nm cutoff filter (CELHXF300-T3, Beijing Zhongjiaojinyuan Co., Ltd., Beijing, China) was utilized. During the bactericidal reactions, 5 mL suspension was withdrawn and centrifugated at different intervals. The supernatant was further used for the determination of extracellular K^+^, DNA, and protein concentration. The precipitate was washed three times with a PBS buffer solution (0.1 mol L^−1^, pH 7.4) and suspended in a 10 mL PBS buffer solution. The above solution (1 mL) was diluted with sterilized saline and incubated onto an agar plate at 37 °C for 48 h. The plate count method was applied to examine the *E. coli* cell density at different irradiation time. A certain amount of *E. coli* solution was diluted with a gradient of 10^−1^ and coated on a flat plate containing an LB solid medium, with incubation at 37 °C for 24 h. The number of colonies on the plate was counted. Laser scanning confocal microscopy (CLSM) and SEM were performed to detect the dead/live *E. coli* cells and the cell morphology. Furthermore, the superoxide dismutase (SOD) and catalase (CAT) activities of *E. coli* were measured.

### 3.5. Fluorescence Microscopy Assays of Live/Dead E. coli

To distinguish live and dead cells, SYTO9 and propidium iodide (PI) were chosen as the staining agents. The PI solution (5 μg/mL) and SYTO9 solution (5 μg/mL) were mixed with an equal volume. The above PI/SYTO9 solution was added into the PBS buffer solution with *E. coli* prepared according to the method described in Section 2.4 and the reaction was conducted in the darkness for 10 min. After centrifugation, the stained *E. coli* was washed with PBS and observed using CLSM.

### 3.6. Microstructure Observation of E. coli

After washing with PBS in the suspension described in Section 2.4, the *E. coli* cells were fixed with a 2.5% (*v*/*v*) glutaraldehyde solution at 4 °C for 6 h. Subsequently, the *E. coli* cells were washed with the PBS buffer solution and gradually dehydrated with an ethanol solution (30%, 50%, 70%, 90%, and 100%) for 10 min each time and with tert-butanol for 20 min. After supercritical drying, the microstructure of the *E. coli* cells was examined using SEM.

### 3.7. Antioxidant Enzymes Assay

The supernatants at various intervals as described in Section 2.4 were employed for CAT and SOD activity assays. The CAT assay kit (A007-1, Jiancheng Biotech, Nanjing, China) and the SOD assay kit (A001-1, Jiancheng Biotech, Nanjing, China) were used according to the instructions.

### 3.8. Leakage of Intracellular Components

The supernatant obtained from the suspension at different irradiation time as described in Section 2.4 was used to study the leakage of K^+^, proteins, and DNA. ICP-OES (5110VDV, Agilent Technologies Co. Ltd., Palo Alto, CA, USA) was operated to detect the K^+^ released from the *E. coli* cells. Extracellular protein content was evaluated by BCA protein quantification kit (PA115, Tiangen Biochemical Technology Co. Ltd., Beijing, China). Extracellular DNA content was determined using a NanoDrop One at 260 nm.

## 4. Conclusions

This work successfully constructed a novel S-scheme CuS/Bi_5_O_7_I heterojunction successfully using a two-step approach comprising the alkaline hydrothermal method and the adsorption–deposition method. Overall, 3%-CuS/Bi_5_O_7_I composite exhibited the optimal disinfection of *E. coli* with visible light illumination, completely inactivating *E. coli* (5 × 10^8^ cfu/mL) within 180 min. The active species, including •O_2_^−^ and h^+^, destroyed the cell membranes of the bacteria. Cellular components, such as K^+^, protein, and DNA, were released as the permeability of the cell membranes changed. The S-scheme transfer pathway for the photoinduced electrons and holes across the CuS/Bi_5_O_7_I heterojunction was revealed. The high separation efficiency of photoinduced carriers was achieved, as well as an enhanced redox capacity, which contributed to improved photocatalytic disinfection activity. This work offers fresh insights into the design of S-scheme Bi_5_O_7_I-based heterojunctions for the inactivation of bacteria utilizing inexhaustible solar energy.

## Data Availability

The data of the study can be provided by corresponding author upon reasonable request.

## Supplementary Materials

The following supporting information can be downloaded at: https://www.mdpi.com/article/10.3390/molecules28073084/s1, Figure S1: XPS survey spectrum of 3%-CuS/Bi_5_O_7_I; Figure S2: Photocatalytic disinfection efficiency of *E. coli* with different scavengers in the dark; Figure S3: Type II charge transfer mechanism of CuS/Bi_5_O_7_I composite against *E. coli* under visible light irradiation; Table S1: Analysis results of EDS.

## Sample Availability

Samples of the compounds are available from the authors.

## References

[1] Matsunaga T., Tomoda R., Nakajima T., Wake H. (1985). Photoelectrochemical sterilization of microbial cells by semiconductor powders. Fems. Microbiol. Lett..

[2] Kumar A., Hasija V., Sudhaik A., Raizada P., Nguyen V.H., Le Q.V., Singh P., Nguyen D.C., Thakur S., Hussain C.M. (2022). The practicality and prospects for disinfection control by photocatalysis during and post-pandemic: A critical review. Environ. Res..

[3] Habibi-Yangjeh A., Asadzadeh-Khaneghah S., Feizpoor S., Rouhi A. (2020). Review on heterogeneous photocatalytic disinfection of waterborne, airborne, and foodborne viruses: Can we win against pathogenic viruses?. J. Colloid Interf. Sci..

[4] Wang Y., Meng J., Jing S., Wang K., Ban C., Feng Y., Duan Y., Ma J., Gan L., Zhou X. (2022). Origin of bismuth-rich strategy in bismuth oxyhalide photocatalysts. Energy Environ. Mater..

[5] Han Q. (2021). Advances in preparation methods of bismuth-based photocatalysts. Chem. Eng. J..

[6] Zhang W., Peng Y., Yang Y., Zhang L., Bian Z., Wang H. (2022). Bismuth-rich strategy intensifies the molecular oxygen activation and internal electrical field for the photocatalytic degradation of tetracycline hydrochloride. Chem. Eng. J..

[7] Yang J., Xu L., Liu C., Xie T. (2014). Preparation and photocatalytic activity of porous Bi_5_O_7_I nanosheets. Appl. Surf. Sci..

[8] Cao C.-S., Wang J., Yu X., Zhang Y., Zhu L. (2020). Photodegradation of seven bisphenol analogues by Bi_5_O_7_I/UiO-67 heterojunction: Relationship between the chemical structures and removal efficiency. Appl. Catal. B Environ..

[9] Bai Y., Ye L., Chen T., Wang L., Shi X., Zhang X., Chen D. (2016). Facet-dependent photocatalytic N_2_ fixation of bismuth-rich Bi_5_O_7_I nanosheets. ACS Appl. Mater. Interf..

[10] Lan M., Zheng N., Dong X., Hua C., Ma H., Zhang X. (2020). Bismuth-rich bismuth oxyiodide microspheres with abundant oxygen vacancies as an efficient photocatalyst for nitrogen fixation. Dalton Trans..

[11] Ding C., Ye L., Zhao Q., Zhong Z., Liu K., Xie H., Bao K., Zhang X. (2016). Huang, synthesis of BixOyIz from molecular precursor and selective photoreduction of CO_2_ into CO. J. CO2 Util..

[12] Li Q., Li Y.-L., Li B., Hao Y.-J., Wang X.-J., Liu R.-H., Ling Y., Liu X., Li F.-T. (2021). Construction of adjustable dominant {314} facet of Bi_5_O_7_I and facet-oxygen vacancy coupling dependent adsorption and photocatalytic activity. Appl. Catal. B Environ..

[13] Lin J., Hu Z., Li H., Qu J., Zhang M., Liang W., Hu S. (2019). Ultrathin nanotubes of Bi_5_O_7_I with a reduced band gap as a high-performance photocatalyst. Inorg. Chem..

[14] Ju P., Hao L., Zhang Y., Sun J., Dou K., Lu Z., Liao D., Zhai X., Sun C. (2023). In-situ topotactic construction of novel rod-like Bi_2_S_3_/Bi_5_O_7_I p-n heterojunctions with highly enhanced photocatalytic activities. J. Mater. Sci. Technol..

[15] Huang W., Xiao X., Lu M., Xiao Y. (2022). In-situ fabrication of novel BiOCl/Bi_5_O_7_I 2D/3D heterostructures with enhanced photocatalytic activity. J. Alloys Compd..

[16] Guan Y., Wu J., Liu Q., Wang H., Liu G., He P., Qi X. (2021). Study of sheetlike BiOI/rodlike Bi_5_O_7_I composite photocatalyst by in situ crystallization of BiOI with pH-dependence for Hg0 removal. Energy Fuels.

[17] Li J., Liu E., Ma Y., Hu X., Wan J., Sun L., Fan J. (2016). Synthesis of MoS_2_/g-C_3_N_4_ nanosheets as 2D heterojunction photocatalysts with enhanced visible light activity. Appl. Surf. Sci..

[18] Zhu Y., Xu J., Chen M. (2022). Synthesis of La_2_Ti_2_O_7_/Bi_5_O_7_I photocatalysts with improved photocatalytic activity for degradation of CIP under visible light. Sep. Purif. Technol..

[19] Fu J., Xu Q., Low J., Jiang C., Yu J. (2019). Ultrathin 2D/2D WO_3_/g-C_3_N_4_ step-scheme H_2_-production photocatalyst. Appl. Catal. B Environ..

[20] Zhang L., Zhang J., Yu H., Yu J. (2022). Emerging S-scheme photocatalyst. Adv. Mater..

[21] Xu Q., Zhang L., Cheng B., Fan J., Yu J. (2020). S-scheme Heterojunction photocatalyst. Chem.

[22] Zhang B., Shi H., Yan Y., Liu C., Hu X., Liu E., Fan J. (2021). A novel S-scheme 1D/2D Bi_2_S_3_/g-C_3_N_4_ heterojunctions with enhanced H_2_ evolution activity. Colloid Surf. A.

[23] Lai C., Zhang M., Li B., Huang D., Zeng G., Qin L., Liu X., Yi H., Cheng M., Li L. (2019). Fabrication of CuS/BiVO_4_ (0 4 0) binary heterojunction photocatalysts with enhanced photocatalytic activity for Ciprofloxacin degradation and mechanism insight. Chem. Eng. J..

[24] Li Z., Zhang Z., Dong Z., Wu Y., Zhu X., Cheng Z., Liu Y., Wang Y., Zheng Z., Cao X. (2021). CuS/TiO_2_ nanotube arrays heterojunction for the photoreduction of uranium (VI). J. Solid State Chem..

[25] Ni L., Wang T., Wang H., Wang Y. (2022). An anaerobic-applicable Bi_2_MoO_6_/CuS heterojunction modified photocatalytic membrane for biofouling control in anammox MBRs: Generation and contribution of reactive species. Chem. Eng. J..

[26] Majhi D., Bhoi Y.P., Samal P.K., Mishra B.G. (2018). Morphology controlled synthesis and photocatalytic study of novel CuS-Bi_2_O_2_CO_3_ heterojunction system for chlorpyrifos degradation under visible light illumination. Appl. Surf. Sci..

[27] Wang J., Cao C., Wang Y., Wang Y., Sun B., Zhu L. (2020). In situ preparation of p-n BiOI@Bi_5_O_7_I heterojunction for enhanced PFOA photocatalytic degradation under simulated solar light irradiation. Chem. Eng. J..

[28] Han X., Wang S., Huang H., Zhang Y. (2021). Hydroxyl radicals and sulfate radicals synergistically boosting the photocatalytic and mineralization ability of 1D-2D Bi_5_O_7_I/NiFe-LDH heterojunction. Appl. Surf. Sci..

[29] Xu C., Yan K., Wang P., Zhou X., Zhang T., Fu Y., Yan Q. (2021). CuBi_2_O_4_ and rGO co-modified 3D hierarchical flower-like Bi_5_O_7_I nanoflakes as Z-scheme heterojunction for enhanced photocatalytic performance. Sep. Purif. Technol..

[30] Dong Z., Pan J., Wang B., Jiang Z., Zhao C., Wang J., Song C., Zheng Y., Cui C., Li C. (2018). The p-n-type Bi_5_O_7_I-modified porous C_3_N_4_ nano-heterojunction for enhanced visible light photocatalysis. J. Alloys Compd..

[31] Das K., Majhi D., Bhoi Y.P., Mishra B.G. (2019). Combustion synthesis, characterization and photocatalytic application of CuS/Bi_4_Ti_3_O_12_ p-n heterojunction materials towards efficient degradation of 2-methyl-4-chlorophenoxyacetic acid herbicide under visible light. Chem. Eng. J..

[32] Shi H., Wan J., Dong X., Xi J., Zhang L., Wang W., Zhang X., Shi Y., Tang Z. (2023). Ag bridged step-scheme MoS_2_/Bi_4_O_5_Br_2_ heterojunction for enhanced visible light driven photocatalytic disinfection activity. Appl. Surf. Sci..

[33] Miao Z., Wang Q., Zhang Y., Meng L., Wang X. (2022). In situ construction of S-scheme AgBr/BiOBr heterojunction with surface oxygen vacancy for boosting photocatalytic CO_2_ reduction with H_2_O. Appl. Catal. B Environ..

[34] Xia C., Lu R., Han Q. (2022). Synthesis of Bi_4_O_5_I_2_/Bi_5_O_7_I heterojunction at weak acidic solution with preferentially growing facets and high photocatalytic activity. Opt. Mater..

[35] Pang J., Han Q., Liu W., Shen Z., Wang X., Zhu J. (2017). Two basic bismuth nitrates: [Bi_6_O_6_(OH)_2_](NO_3_)_4_·2H_2_O with superior photodegradation activity for rhodamine B and [Bi_6_O_5_(OH)_3_](NO_3_)_5_·3H_2_O with ultrahigh adsorption capacity for methyl orange. Appl. Surf. Sci..

[36] Lu C., Wang L., Yang D., Jin Z., Wang X., Xu J., Li Z., Shi W., Guan W., Huang W. (2022). Boosted tetracycline and Cr(VI) simultaneous cleanup over Z-Scheme BiPO_4_/CuBi_2_O_4_ p-n heterojunction with 0D/1D trepang-like structure under simulated sunlight irradiation. J. Alloys Compd..

[37] Li J., Yang R., Hu D., Xu Y., Ma Z. (2022). Efficient bacterial inactivation with S-doped g-C_3_N_4_ nanosheets under visible light irradiation. Environ. Sci. Pollut. R.

[38] Du J., Xu Z., Li H., Yang H., Xu S., Wang J., Jia Y., Ma S., Zhan S. (2021). Ag_3_PO_4_/g-C_3_N_4_ Z-scheme composites with enhanced visible-light-driven disinfection and organic pollutants degradation: Uncovering the mechanism. Appl. Surf. Sci..

[39] Shi H., Xie Y., Wang W., Zhang L., Zhang X., Shi Y., Fan J., Tang Z. (2022). In-situ construction of step-scheme MoS_2_/Bi_4_O_5_Br_2_ heterojunction with improved photocatalytic activity of Rhodamine B degradation and disinfection. J. Colloid Interf. Sci..

[40] Qi Z., Li G., Wang M., Chen C., Xu Z., An T. (2022). Photoelectrocatalytic inactivation mechanism of *E. coli* DH5alpha (TET) and synergistic degradation of corresponding antibiotics in water. Water Res..

[41] Ming J., Sun X., Ma Q., Liu N., Zhang C., Kawazoe N., Chen G., Yang Y. (2023). Advanced photocatalytic sterilization for recalcitrant Enterococcus sp. contaminated water by newly developed Z-scheme Bi_2_WO_6_ based composites under solar light. Chemosphere.

[42] Diao M., Qi D., Xu M., Lu Z., Lv F., Bie X., Zhang C., Zhao H. (2018). Antibacterial activity and mechanism of monolauroyl-galactosylglycerol against *Bacillus cereus*. Food Control.

[43] Guan G., Zhang L., Zhu J., Wu H., Li W., Sun Q. (2021). Antibacterial properties and mechanism of biopolymer-based films functionalized by CuO/ZnO nanoparticles against *Escherichia coli* and *Staphylococcus aureus*. J. Hazard Mater..

[44] Xia D., Liu H., Xu B., Wang Y., Liao Y., Huang Y., Ye L., He C., Wong P.K., Qiu R. (2019). Single Ag atom engineered 3D-MnO_2_ porous hollow microspheres for rapid photothermocatalytic inactivation of *E. coli* under solar light. Appl. Catal. B Environ..

[45] Xiao K., Wang T., Sun M., Hanif A., Gu Q., Tian B., Jiang Z., Wang B., Sun H., Shang J. (2020). Photocatalytic Bacterial inactivation by a rape pollen-MoS_2_ Biohybrid catalyst: Synergetic effects and inactivation mechanisms. Environ. Sci. Technol..

[46] Shi W., Li M., Huang X., Ren H., Guo F., Tang Y., Lu C. (2020). Construction of CuBi_2_O_4_/Bi_2_MoO_6_ p-n heterojunction with nanosheets-on-microrods structure for improved photocatalytic activity towards broad-spectrum antibiotics degradation. Chem. Eng. J..

[47] Fan H.-T., Wu Z., Liu K.-C., Liu W.-S. (2022). Fabrication of 3D CuS@ZnIn_2_S_4_ hierarchical nanocages with 2D/2D nanosheet subunits p-n heterojunctions for improved photocatalytic hydrogen evolution. Chem. Eng. J..

[48] Li S., Cai M., Liu Y., Zhang J., Wang C., Zang S., Li Y., Zhang P., Li X. (2022). In situ construction of a C_3_N_5_ nanosheet/Bi_2_WO_6_ nanodot S-scheme heterojunction with enhanced structural defects for the efficient photocatalytic removal of tetracycline and Cr(vi). Inorg. Chem. Front..

[49] Jiang Y., Wang Y., Zhang Z., Dong Z., Xu J. (2022). 2D/2D CsPbBr_3_/BiOCl heterojunction with an S-scheme charge transfer for boosting the photocatalytic conversion of CO_2_. Inorg. Chem..

[50] Yang H., He D., Liu C., Zhang T., Qu J., Jin D., Zhang K., Lv Y., Zhang Z., Zhang Y.N. (2022). Visible-light-driven photocatalytic disinfection by S-scheme alpha-Fe_2_O_3_/g-C_3_N_4_ heterojunction: Bactericidal performance and mechanism insight. Chemosphere.

[51] Huang Z.-F., Song J., Wang X., Pan L., Li K., Zhang X., Wang L., Zou J.-J. (2017). Switching charge transfer of C_3_N_4_/W_18_O_49_ from type-II to Z-scheme by interfacial band bending for highly efficient photocatalytic hydrogen evolution. Nano Energy.

[52] Chen Q., Lan X., Chen K., Ren Q., Shi J. (2022). Construction of WO_3_/CsPbBr_3_ S-scheme heterojunction via electrostatic Self-assembly for efficient and Long-Period photocatalytic CO_2_ reduction. J. Colloid Interf. Sci..

[53] Luo J., Zhou X., Yang F., Ning X., Zhan L., Wu Z., Zhou X. (2022). Generating a captivating S-scheme CuBi_2_O_4_/CoV_2_O_6_ heterojunction with boosted charge spatial separation for efficiently removing tetracycline antibiotic from wastewater. J. Clean. Prod..

