# Supplementary material for: Construction of S-Scheme CuS/Bi5O7I Heterojunction for Boosted Photocatalytic Disinfection with Visible Light Exposure
Source: Molecules. 2023 Mar 30;28(7):3084. doi: 10.3390/molecules28073084 (PMC10096083; doi:10.3390/molecules28073084)
Supplement: Supplementary file 1 [file molecules-28-03084-s001.zip › molecules-2277154-supplementary.pdf]

Supporting Information

Construction of S-Scheme CuS/Bi_5O_7I Heterojunction for Boosted Photocatalytic Disinfection with Visible Light Exposure

Zhanqiang Ma [1,*], Wei Guo [1], Kaiyue Zhang [1], Nan Wang [1], Ziyue Li [1] and Juan Li [2,*]

[1] College of Agriculture, Henan University of Science and Technology, Luoyang 471000, China; gw18438616338@163.com (W.G.); zky245385286@163.com (K.Z.); wangn0324@163.com (N.W.); liziyue667788@163.com (Z.L.)
[2] School of Environmental Engineering and Chemistry, Luoyang Institute of Science and Technology, Luoyang 471023, China
* Correspondence: mzqnxy@163.com (Z.M.), lijuan_ly@163.com (J.L.)

Chemicals and reagents

$Bi(NO_3)_3 \cdot 5H_2O$, KI, NaOH, $Cu(CH_3COO)_2 \cdot H_2O$, K_2S, ammonium oxalate (AO), isopropanol (IPA) and benzoquinone (BQ) were purchased from Shanghai Macklin Biochemical Co., Ltd, China. The above reagents were analytical grade. Tryptone, agar and yeast extract used for Luria-Bertani (LB) medium were provided from Shanghai Aladdin Biochemical Technology Co., Ltd., China.

Characterization

SEM images were obtained by JEOL JSM-IT200 scanning electron microscope and the EDS coupled with the scanning electron microscopy. Powder X-ray diffraction (XRD) of the samples was carried out on Bruker D8A X-ray powder diffractometer with Cu Kα radiation operating at 40 kV and 40 mA. X-ray photoelectron spectroscopy (XPS) measurements were performed by Kratos AXIS NOVA spectrometer. UV-vis diffuse reflectance spectra (DRS) were obtained from Shimadzu UV-2600i spectrophotometer. Photoluminescence (PL) spectra were measured on Hitachi-F7000 fluorescence spectrophotometer.

Photoelectrochemical measurement

The electrochemical tests were studied on CHI760E electrochemical workstation using a three-electrode quartz reactor. 20 mg sample were coated on the fluorine-doped tin oxide (FTO) glass as working electrode. Ag/AgCl electrode and Pt wire were taken as reference electrode and counter electrode, respectively. 0.2 M Na_2SO_4 solution was selected as the supporting electrolyte for photoelectrochemical experiments. Besides, transient photocurrent response tests and electrochemical impedance spectroscopy (EIS) were performed under 300W Xe lamp irradiation. The Mott-Schottky (M-S) plots were measured in the dark. Moreover, EIS was determined in a frequency range from 1 Hz to 100 kHz and M-S plots were studied at the frequency of 1000 Hz.

Table S1. Analysis results of EDS

Element	Weight (%)	Atomic (%)
C	32.68±0.23	71.50±0.50
O	12.55±0.31	20.61±0.51
S	0.37±0.05	0.31±0.04
Cu	1.06±0.12	0.44±0.05
I	5.39±0.20	1.12±0.04
Bi	47.96±0.57	6.03±0.07

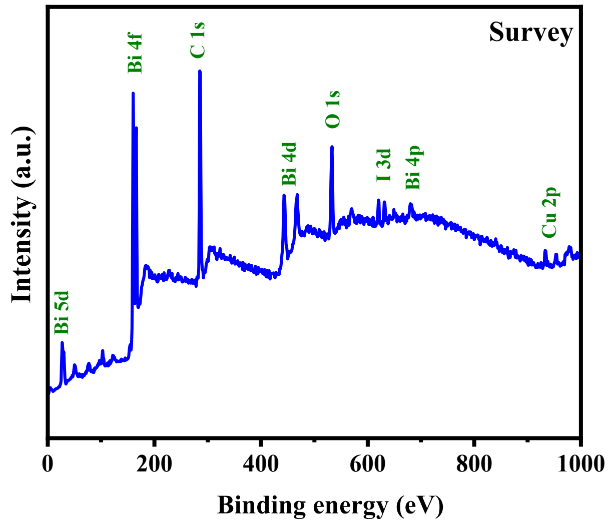

Figure S1. XPS survey spectrum of 3%-CuS/Bi_5O_7I.

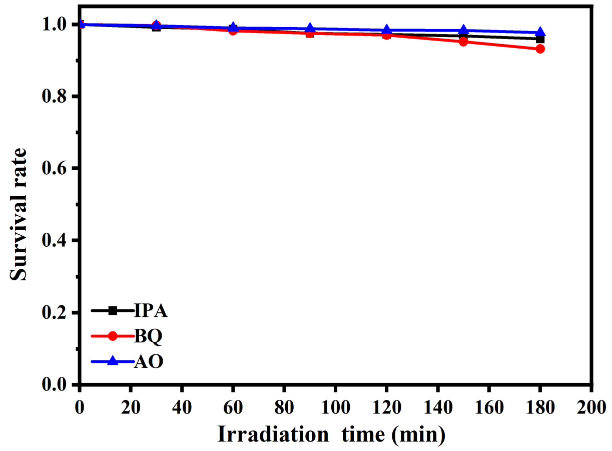

Figure S2. Photocatalytic disinfection efficiency of *E. coli* with different scavengers in the dark.

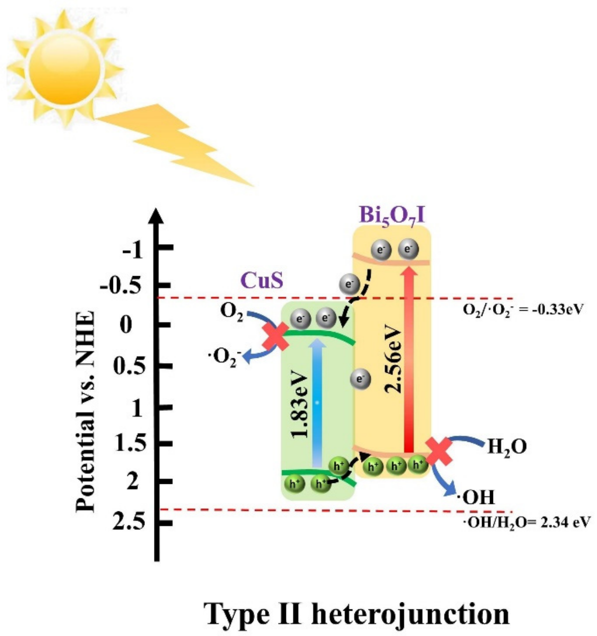

Figure S3. Type Ⅱ charge transfer mechanism of CuS/Bi_5O_7I composite against *E. coli* under visible light irradiation.
